# The anterior scleral thickness in primary open-angle glaucoma with high myopia

**DOI:** 10.3389/fmed.2024.1356839

**Published:** 2024-06-28

**Authors:** Mu Li, Liwen Chen, Zhaoxia Luo, Xiaoqin Yan

**Affiliations:** ^1^Department of Ophthalmology, Wuhan Union Hospital, Tongji Medical College, Huazhong University of Science and Technology, Wuhan, China; ^2^Department of Ophthalmology, Tongji Hospital, Tongji Medical College, Huazhong University of Science and Technology, Wuhan, China

**Keywords:** primary open-angle glaucoma, high myopia, anterior scleral thickness, trabecular meshwork, Schlemm’s canal

## Abstract

**Purpose:**

This study aimed to investigate and compare the anterior scleral thickness (AST) among high myopia (HM), primary open-angle glaucoma (POAG), and POAG with HM (HMPOAG) groups.

**Methods:**

Thirty-two HM eyes, 30 POAG eyes, and 31 HMPOAG eyes were included. The Schlemm’s canal (SC) area, trabecular meshwork (TM) thickness, scleral spur (SS) length, and AST were measured using swept-source optical coherence tomography. AST was measured at 0 mm (AST0), 1 mm (AST1), 2 mm (AST2), and 3 mm (AST3) from SS.

**Results:**

The HMPOAG group had significantly thinner AST, SS length, and TM thickness than the HM and POAG groups (all *p* < 0.05). In addition, the SC area of the HMPOAG group was also significantly smaller than that of the HM group (*p* < 0.001).

**Conclusion:**

The HMPOAG group had the thinnest AST, shortest SS, thinnest TM, and smallest SC. The thinnest AST might contribute to the shortest SS, and further to the thinnest TM and smallest SC in the HMPOAG group. AST might be a novel clinical indicator in the prediction and evaluation of POAG.

## Introduction

Myopia is suggested to be an important risk factor for primary open-angle glaucoma (POAG) (1–3). For subjects with myopia, the risk of developing POAG was twice as high as for those without myopia. Furthermore, high myopia (HM) could increase the risk of developing POAG to approximately six times that of non-myopia subjects (1, 4). Moreover, the prevalence of open-angle glaucoma (OAG) is positively associated with increasing myopia, and glaucomatous optic neuropathy is more obvious in eyes with moderate and HM (5, 6). Both the HM and POAG groups exhibit similar changes in scleral collagen and hypersensitive responses to glucocorticoids (7). Besides that, previous studies have suggested that POAG with HM (HMPOAG) patients have greater and more significant progression of visual field loss (8, 9). Therefore, HM may be an independent risk factor for OAG (6, 10, 11).

The major resistance to aqueous humor outflow is reported to be the juxtacanalicular tissue of the trabecular meshwork (TM) and the inner wall of Schlemm’s canal (SC), and the open status of TM and SC is crucial for the outflow of aqueous humor (12–14). The sclera influences the biomechanical environment of ocular tissues (15) and supports more delicate intraocular structures (e.g., the cornea) (16). The TM and SC are situated in the limbus, which is right between the cornea and anterior sclera (17, 18). Previous studies have highlighted the significance of anterior scleral thickness (AST) in maintaining the morphology of TM and SC (17). Moreover, the scleral spur (SS), part of the anterior sclera, is considered essential in maintaining the patency of TM and SC and facilitating aqueous humor outflow. A shorter SS may risk developing POAG due to insufficient support for TM and SC morphology, whereas a longer SS can better maintain TM and SC morphology (19–21). Although studies have shown AST and SS lengths in POAG (17, 19) and HM (18, 22) eyes, no study has yet investigated changes in the morphology of the anterior sclera, SS, TM, and SC in HMPOAG patients. Accordingly, in this study, we used swept-source optical coherence tomography (SS-OCT) to measure AST, SS length, TM thickness, and SC area, and retinal nerve fiber layer (RNFL) thickness in patients with HM, POAG, and HMPOAG, respectively.

## Materials and methods

This study was approved by the ethics committee of Tongji Hospital, Huazhong University of Science and Technology (TJ-IRB20201024), and adhered to the tenets of the Declaration of Helsinki. All subjects signed written informed consent before study participation.

### Study subjects

Thirty-two HM eyes from 32 HM subjects, 30 POAG eyes from 30 POAG subjects, and 31 HMPOAG eyes from 31 HMPOAG subjects were included in this study. Ophthalmic examinations, including axial length (AL) measurement (IOL-Master 500, Carl Zeiss Meditec, Dublin, United States), refractive error measurement (RT-2100, NIDEK Co. Ltd., Gamagori, Japan), central corneal thickness (CCT) measurement (corneal map, CASIA SS-1000, Tomey Corp., Nagoya, Japan), intraocular pressure (IOP) measurement, fundus photography, slit-lamp examination, gonioscopy, standard automated perimetry examination (Humphrey Field Analyzer, Carl Zeiss Meditech, Dublin, United States), and RNFL thickness measurement (spectral domain-OCT, Heidelberg Engineering GmbH, Heidelberg, Germany), were performed. POAG was defined as a glaucomatous-appearing optic nerve, RNFL defect, glaucomatous visual field defects corresponding to optic nerve changes, and normal anterior chamber depth with an open angle (17). HM was defined as spherical equivalent (SE) less than −6.00 diopter (D) or AL ≥ 26.0 mm (18). Subjects with a history of scleritis, a history of uveitis, prior ocular surgery, prior ocular trauma, or systemic disease were excluded from participation. One eye was randomly selected from each subject.

### SS-OCT imaging acquisition and processing

The recruited eyes were imaged using a high-density scan of SS-OCT. Subjects were instructed to open their eye wide during scanning. The nasal and temporal limbi were recorded separately after adjusting the scan fixture to the corresponding areas. The scan line was horizontal without any rotation. Each eye was scanned three times, and the best quality image would be selected for ocular biometric measurements.

### Measurements of SC area, TM thickness, SS length, and AST

**Figure 1:**
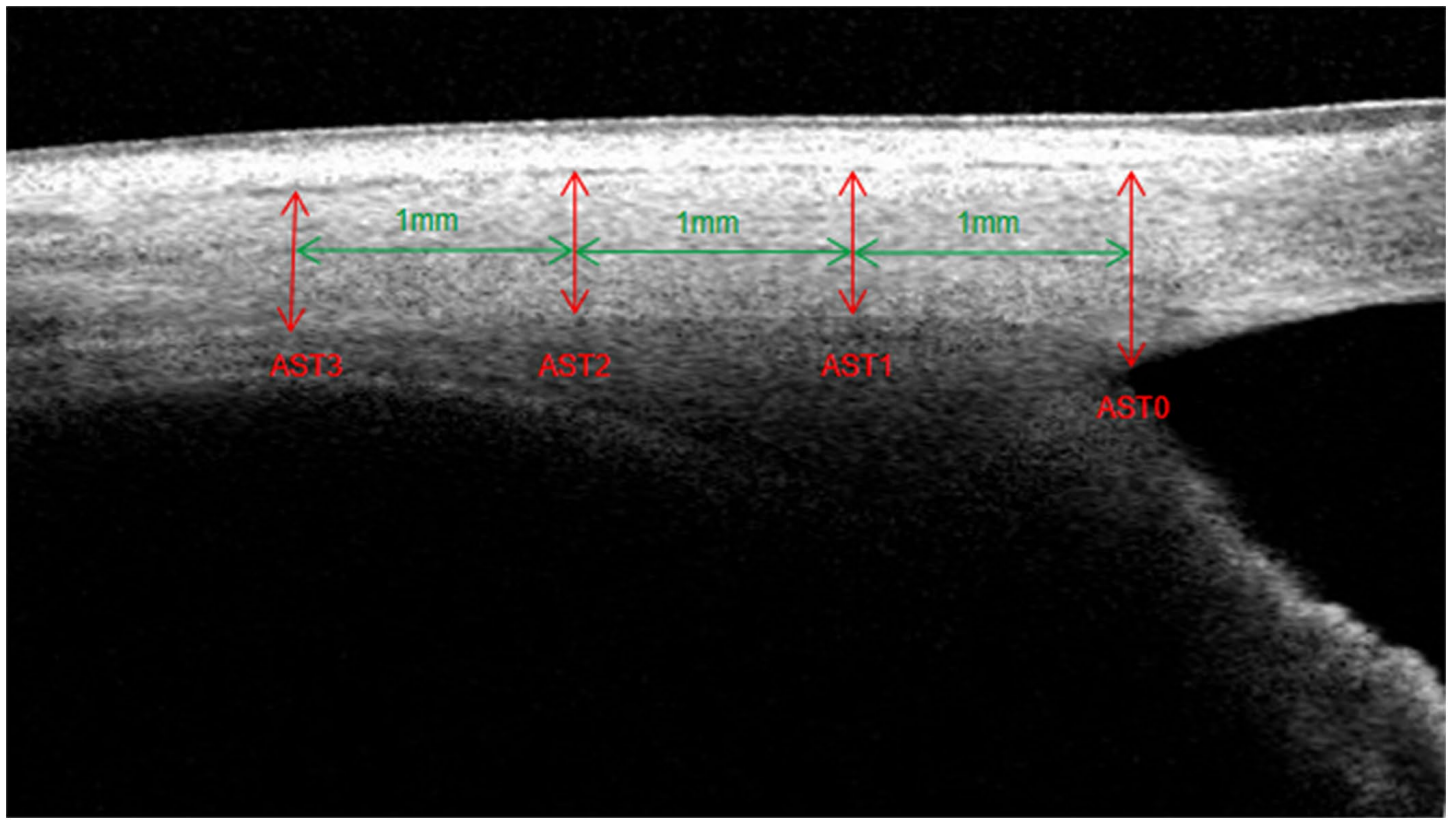
Measurements of anterior scleral thickness (AST0, AST1, AST2, and AST3).

The optimal image magnification, contrast, and brightness were subjectively defined to maximize the visualization of the ocular anterior segment biometrics. The SC was defined as a thin, black, lucent space in the OCT image, and the SC area was manually drawn freehand based on the outline of the SC (19, 23, 24). TM thickness was measured as the average value of two measurements made at the halfway point and anterior point of the SC inner wall. Each TM thickness measurement was perpendicular to the TM inner layer, beginning from the SC inner wall (25, 26). SS length was defined as the line bisecting the width of the SS at every point, starting from the tip of SS to the midpoint of the anterior and posterior points where the sclera curves out to form the spur (19). AST was the distance from the episcleral blood vessels (a thin hyporeflective area in the anterior part of the sclera) to the posterior boundary of the sclera (the line separating the hyperreflective sclera from the hyporeflective ciliary muscle) (17, 18, 22). AST was measured at 0 mm (AST0), 1 mm (AST1), 2 mm (AST2), and 3 mm (AST3) from SS (Figure 1). All measurements were performed using ImageJ software (National Institutes of Health, Bethesda, MD, United States), and the observer was masked to the subject’s information.

### Statistical analyses

All analyses were conducted by R software version 3.4.3^1^ and EmpowerStats software (www.empowerstats.com, X&Y Solutions, Inc., Boston, MA, United States). The data were shown as mean ± standard deviation where applicable. The Kruskal–Wallis H-test was used for intergroup comparisons of age, AL, SE, CCT, mean deviation (MD) of the visual field, and RNFL thickness, whereas the chi-square statistic was used for intergroup comparisons of sex. Generalized estimating equations (GEEs), which take the correlation of nasal and temporal measurements of one eye into account, were used for the intergroup comparisons of the SC area, TM thickness, SS length, and AST (27, 28). Linear regression was used to determine the associations between the SC area, TM thickness, and AST, as well as the associations between SS length and AST and RNFL thickness. Adjusted β coefficients for the associations between independent and dependent variables were assessed using GEEs. The intra-observer and inter-observer reproducibility of ocular anterior segment biometrics measurements were assessed using the intraclass correlation coefficient (ICC) with a 95% confidence interval (CI). All tests were two-tailed, and a *p*-value of <0.05 was considered statistically significant.

## Results

As shown in Table 1, there were no significant differences in age, sex, or CCT among the HM, POAG, and HMPOAG groups (all *p* > 0.05). AL was significantly shorter, and SE was significantly greater in the POAG group than in the HM and HMPOAG groups (all *p* < 0.001). IOP was significantly lower in the HM group than in the POAG and HMPOAG groups (both *p* < 0.001). MD of visual field and RNFL thickness was significantly greater in the HM group than in the POAG and HMPOAG groups (all *p* < 0.001).

**Table 1:** Intergroup comparisons of age, sex, AL, SE, CCT, IOP, MD of visual field, and RNFL thickness.

	HM (*N* = 32)	POAG (*N* = 30)	HMPOAG (*N* = 31)	*p1*	*p2*	*p3*
Age (years)^a^ (range)	35.16 ± 13.39 (22 to 64)	39.80 ± 12.10 (24 to 59)	35.65 ± 8.77 (23 to 55)	0.095	0.864	0.277
Sex (M/F)^b^	15/17	18/12	19/12	0.322	0.315	1.000
AL (mm)^a^ (range)	26.66 ± 0.98 (25.3 to 29.3)	23.09 ± 0.90 (21.2 to 24.8)	26.94 ± 1.13 (25.1 to 31)	<0.001^*^	1.000	<0.001^*^
SE (D)^a^ (range)	−7.99 ± 2.40 (−14 to −6)	−0.51 ± 1.41 (−3 to 1.5)	−7.76 ± 1.75 (−11.5 to −6)	<0.001^*^	1.000	<0.001^*^
CCT (μm)^a^ (range)	536.31 ± 26.36 (494 to 629)	534.77 ± 28.21 (479 to 591)	535.61 ± 33.38 (460 to 613)	1.000	1.000	1.000
IOP (mmHg)^a^ (range)	15.36 ± 2.12 (10.7 to 19)	20.77 ± 5.43 (11 to 33)	22.58 ± 7.66 (15 to 48)	<0.001^*^	<0.001^*^	1.000
MD (dB)^a^ (range)	−1.41 ± 1.11 (−4.65 to 1.06)	−13.18 ± 8.61 (−30.18 to −1.48)	−12.50 ± 6.84 (−28.85 to −3.69)	<0.001^*^	<0.001^*^	1.000
RNFL (μm)^a^ (range)	86.81 ± 4.09 (80 to 96)	56.67 ± 13.57 (35 to 78)	57.58 ± 12.16 (31 to 73)	<0.001^*^	<0.001^*^	1.000

HM: high myopia; POAG: primary open-angle glaucoma; M: male; F: female; AL: axial length; SE: spherical equivalent; CCT: central corneal thickness; IOP: intraocular pressure; MD: mean deviation; RNFL: retinal nerve fiber layer. p1: the comparisons between HM and POAG groups; p2: the comparisons between HM and HMPOAG groups; p3: the comparisons between POAG and HMPOAG groups.

a Kruskal–Wallis H-test with Bonferroni adjustment for *p*-values.

b Chi-square statistic. *Statistical significance.

As shown in Table 2, the SC areas of the POAG and HMPOAG groups were significantly smaller than those of the HM group (both *p* < 0.001). TM thickness was significantly thinner in the HMPOAG group than in the HM and POAG groups (both *p* < 0.05). SS length and AST0 were significantly different among the three groups (all *p* < 0.05). AST1, AST2, and AST3 of the HMPOAG group were significantly thinner than those of the HM and POAG groups (all *p* < 0.05).

**Table 2:** Intergroup comparisons of SC area, TM thickness, SS length, and AST parameters.

	HM (*N* = 32)	POAG (*N* = 30)	HMPOAG (*N* = 31)	*p1*	*p2*	*p3*
SCA (μm^2^) (range)	6636.33 ± 1129.48 (4,253 to 9,061)	5121.47 ± 1370.02 (2,959 to 8,506)	5198.84 ± 985.78 (2,929 to 6,842)	<0.001^*^	<0.001^*^	0.773
TMT (μm) (range)	99.71 ± 34.24 (52.5 to 230)	95.33 ± 23.99 (47 to 157.5)	85.38 ± 19.83 (38.5 to 139.5)	0.438	0.010^*^	0.030^*^
SSL (μm) (range)	209.70 ± 52.36 (132 to 351)	182.98 ± 38.33 (100 to 268)	160.24 ± 43.18 (94 to 312)	0.004^*^	<0.001^*^	0.011^*^
AST0(μm) (range)	707.48 ± 76.73 (583 to 932)	662.65 ± 64.61 (510 to 827)	613.42 ± 60.70 (471 to 760)	0.002^*^	<0.001^*^	<0.001^*^
AST1(μm) (range)	516.43 ± 58.83 (372 to 688)	515.35 ± 59.93 (401 to 702)	467.84 ± 48.83 (375 to 583)	0.932	<0.001^*^	<0.001^*^
AST2(μm) (range)	535.87 ± 57.02 (426 to 707)	530.38 ± 59.51 (375 to 644)	485.27 ± 63.96 (381 to 679)	0.658	<0.001^*^	0.001^*^
AST3(μm) (range)	550.97 ± 56.80 (457 to 716)	535.92 ± 60.46 (413 to 682)	494.06 ± 78.81 (339 to 704)	0.197	<0.001^*^	0.006^*^

HM: high myopia; POAG: primary open-angle glaucoma; SCA: Schlemm’s canal area; TMT: trabecular meshwork thickness; SSL: scleral spur length; AST: anterior scleral thickness. Generalized estimating equations. p1: the comparisons between HM and POAG groups; p2: the comparisons between HM and HMPOAG groups; p3: the comparisons between POAG and HMPOAG groups. *Statistical significance.

### Associations between the SC area, TM thickness, and AST

SC area was significantly associated with AST0, AST1, AST2, and AST3, and TM thickness was significantly associated with AST0, AST1, and AST2 in the HM group (all *p* < 0.05). In both POAG and HMPOAG groups, no significant associations between SC area, TM thickness, and AST were found (all *p* > 0.05) (Table 3).

**Table 3:** Associations between SC area, TM thickness, and AST.

	AST0		AST1		AST2		AST3	
	*β*	*p*	*β*	*p*	*β*	*P*	*Β*	*p*
	HM							
SCA	0.025	0.003^*^	0.017	0.004^*^	0.015	0.001^*^	0.015	0.007^*^
TMT	0.946	0.001^*^	0.377	0.025^*^	0.493	0.007^*^	0.388	0.086
	POAG							
SCA	−0.004	0.681	−0.003	0.696	−0.013	0.106	−0.003	0.768
TMT	0.084	0.626	−0.472	0.286	−0.045	0.869	−0.001	0.999
	HMPOAG							
SCA	0.010	0.185	0.008	0.233	0.008	0.288	0.011	0.086
TMT	0.501	0.186	−0.186	0.528	−0.208	0.510	−0.104	0.748

HM: high myopia; POAG: primary open-angle glaucoma; SCA: Schlemm’s canal area; TMT: trabecular meshwork thickness; AST: anterior scleral thickness. *β/p* value: the influence factors such as age, sex, axial length, spherical equivalent, and central corneal thickness have been adjusted by generalized estimating equations. *Statistical significance.

### Associations between SS length and AST

SS length showed significant associations with all AST parameters in the HM group (all *p* < 0.05), while no significant associations between SS length and AST were found in both POAG and HMPOAG groups (all *p* > 0.05) (Table 4).

**Table 4:** Associations between SS length and AST.

	AST0		AST1		AST2		AST3	
	*β*	*p*	*β*	*p*	*β*	*P*	*Β*	*p*
	HM							
SSL	0.770	<0.001^*^	0.404	<0.001^*^	0.391	0.001^*^	0.390	0.002^*^
	POAG							
SSL	0.280	0.210	−0.013	0.950	0.006	0.979	0.201	0.423
	HMPOAG							
SSL	0.226	0.196	−0.069	0.627	−0.033	0.841	0.023	0.893

HM: high myopia; POAG: primary open-angle glaucoma; SSL: scleral spur length; AST: anterior scleral thickness. *β/p* value: the influence factors such as age, sex, axial length, spherical equivalent, and central corneal thickness have been adjusted by generalized estimating equations. *Statistical significance.

### Associations between RNFL thickness and AST

RNFL thickness had significant and positive associations with all AST parameters in the HMPOAG group (all *p* < 0.05). In both the HM and POAG groups, no significant associations between RNFL thickness and AST were found (all *p* > 0.05) (Table 5).

**Table 5:** Associations between RNFL thickness and AST.

	AST0		AST1		AST2		AST3	
	*β*	*p*	*β*	*p*	*β*	*P*	*Β*	*p*
	HM							
RNFL	1.108	0.789	1.847	0.575	0.102	0.972	0.832	0.746
	POAG							
RNFL	0.214	0.767	0.513	0.443	0.852	0.147	0.765	0.161
	HMPOAG							
RNFL	1.651	0.003^*^	1.109	0.022^*^	2.191	0.003^*^	2.610	0.005^*^

HM: high myopia; POAG: primary open-angle glaucoma; RNFL: retinal nerve fiber layer; AST: anterior scleral thickness. *β/p* value: the influence factors such as age, sex, axial length, spherical equivalent, and central corneal thickness have been adjusted by generalized estimating equations. *Statistical significance.

### The reproducibility of the measurements of ocular parameters

To evaluate the reproducibility of the measurements of ocular parameters, 15 eyes from the HM group, 15 eyes from the POAG group, and 15 eyes from the HMPOAG group were randomly selected. The intra-observer and inter-observer agreements were assessed by ICC (two-way mixed effects, absolute agreement, and single measurement). The ICC values of intra-observer ranged from 0.851 to 0.919, and the ICC values of inter-observer ranged from 0.826 to 0.909 in this study (Table 6).

**Table 6:** Intra-observer and inter-observer reproducibility of the measurements of ocular parameters.

	ICC	Lower	Upper
Intra-observer		95% CI	
SC area (μm^2^)	0.856	0.789	0.903
TM thickness (μm)	0.851	0.782	0.899
SS length (μm)	0.886	0.831	0.923
AST0 (μm)	0.919	0.880	0.946
AST1 (μm)	0.918	0.878	0.945
AST2 (μm)	0.911	0.867	0.940
AST3 (μm)	0.909	0.865	0.939
Inter-observer		95% CI	
SC area (μm^2^)	0.839	0.765	0.891
TM thickness (μm)	0.826	0.747	0.882
SS length (μm)	0.872	0.812	0.914
AST0 (μm)	0.897	0.847	0.931
AST1 (μm)	0.905	0.858	0.936
AST2 (μm)	0.909	0.864	0.939
AST3 (μm)	0.905	0.859	0.936

ICC: intraclass correlation coefficient; CI: confidence interval; SC: Schlemm’s canal; TM: trabecular meshwork; SS: scleral spur; AST: anterior scleral thickness.

## Discussion

The sclera, which accounts for approximately 90% of the outer wall of the eye, is a remarkably resilient and structurally complex connective tissue. It performs various functions critical to vision and provides a tough, fibrous supporting substrate for the ocular components, including the TM, SC, retina, and optic nerve head (16, 17, 19, 20, 29). Changes in scleral properties could affect the ocular mechanical status and biomechanical response to IOP (17, 29), making scleral properties an important factor in glaucoma development and treatment (17, 30). We know from myopic eyes that the thinning of the sclera and changes in the composition of the collagen in the sclera affect the IOP-related behavior of myopic eyes. Thus, the increased risk for glaucoma in myopic eyes may be partly related to the sclera’s mechanical properties (20, 31). Moreover, myopia was also suggested to have decreased retinal and choroidal blood supply (32, 33), which was unbeneficial for neuroprotection in glaucomatous neuropathy. HM and POAG were found to have similar scleral collagen changes and hypersensitive responses to glucocorticoids (7). The prevalence of OAG increased with myopia. Glaucomatous optic neuropathy is more obvious in eyes with moderate to HM, and the visual field defect progressed more rapidly in HMPOAG eyes (5, 6, 8, 9). Thus, myopia is suggested to be a risk factor for POAG (1–3).

Recent studies have shown the importance of the anterior sclera in the pathogenesis of glaucoma, indicating that AST could be an important structural parameter in maintaining TM and SC morphology and plays a role in the outflow of aqueous humor and maintenance of IOP (7, 17). Although changes in AST have previously been reported in the POAG and HM groups (17, 18), the biomechanical characteristics of the anterior sclera in HMPOAG eyes are still unknown.

In this study, we compared AST, SS length, TM, and SC dimensions in the HM, POAG, and HMPOAG groups. We found that AST0 and SS lengths showed identical changing trends, with the thickest AST0 and longest SS lengths in the HM group and the thinnest AST0 and shortest SS lengths in the HMPOAG group. The AST0 and SS lengths of the POAG group were in between (greater than those of the HMPOAG group and less than those of the HM group). Previous studies have indicated that the AST0 and SS lengths of HM eyes were similar to those of normal controls (18), while the AST0 and SS lengths of POAG eyes were smaller than those of normal controls (17). Thus, the AST0 and SS lengths of HM eyes were supposed to be greater than those of POAG eyes, which was consistent with our study results. Furthermore, when combined POAG and HM together in this study, the values of AST0 and SS length would further decrease, with the thinnest AST0 and shortest SS length in the HMPOAG group. Similarly, other anterior scleral parameters (AST1, AST2, and AST3) were also thinnest in the HMPOAG group. A recent study comparing AST in control, HM, and HM with glaucoma (HMG) groups also showed that the AST was significantly thinner in the HMG group than in the control and HM groups (34), which was consistent with our study results.

The TM and SC are situated in the limbus (next to the SS), and the limbus also contains scleral elements (35). SS is a shelf-like structure formed from a projection of the sclera. It is directly attached to the TM and connected to the SC by connecting fibers. By pulling the SS posteriorly, the ciliary muscle can transmit its force to the TM and SC, facilitating aqueous humor outflow by separating the trabecular beams and expanding the SC lumen (20, 25, 36–38). Previous studies suggested that the SS was a supportive tissue for TM and SC. A thicker anterior sclera and a longer SS can better maintain the morphology of TM and SC, keep the open status of TM and SC, and reduce the outflow resistance of aqueous humor (17). When the SS was not long enough, it would be unable to maintain the morphology of TM and SC, resulting in the collapse of TM and SC and the pathogenesis of POAG (17, 19, 21). In addition, the findings that the posterior part of SC where SS exerts the most force is wider than the anterior part of SC were also testament to this function of SS in keeping SC morphology (39). SS belongs to the anterior sclera, and AST (especially AST0, which was the scleral thickness at SS) could have an impact on SS length and further affect the morphology of TM and SC (17). Thus, AST and SS lengths play a critical role in maintaining TM and SC morphology, which is important for the maintenance of IOP (12–14).

In terms of TM thickness, previous studies have indicated that the TM thickness of both the HM and POAG groups was thinner than that of normal controls (17, 18). For the HM group, the excessive elongation of the eyeball of HM could stretch TM, resulting in a decrease in TM thickness (18). For the POAG group, its AST and SS lengths were significantly smaller than those of normal controls (17). Considering the close relationship between AST, SS length, and TM thickness mentioned above, the thinner anterior sclera observed in the POAG group might result in a shorter SS length, further leading to insufficient support for the patency of TM and elevation in IOP (17, 19, 21, 38). On the other hand, the elevated IOP could further compress TM, making TM thinner (13). In this study, we observed that AST was thinnest and SS length was shortest in the HMPOAG group, making the sclera insufficient to maintain the patency of TM, leading to the thinnest TM thickness in the HMPOAG group. Thus, we speculated that HM might aggravate the thinning of glaucomatous TM, making the TM of the HMPOAG group thinner than the POAG group. Compressed/thinner TM might have increased aqueous humor outflow resistance. Previous studies have observed that TM was thinner in POAG eyes than in normal eyes (40). When TM expanded with the administration of Y27632, the aqueous humor outflow facility was reported to be promoted (41). Accordingly, the aqueous humor outflow resistance of HMPOAG eyes was supposed to be the highest among the three study groups (HM, POAG, and HMPOAG groups).

In terms of the SC area, HM eyes were reported to have a larger SC (18, 34), while POAG eyes were reported to have a smaller SC than normal controls (17). In the HM group, the compressed and deformed eyeball might induce obstruction and/or lesions of intra-scleral collector channels and deep scleral plexus, making the resistance distal to SC increase, leading to the expansion of SC lumen (18). In the POAG group, the collapse of SC could partly be attributed to the smaller size of the anterior sclera and SS, which were unable to support SC morphology (17, 19, 21). When combined POAG and HM groups, the SC area of the HMPOAG group was similar to that of the POAG group and was significantly smaller than that of the HM group. Even though HM eyes had SC lumen enlargement effects (18), HMPOAG eyes had a similar SC area to the POAG group (collapsed SC), indicating that the effect of POAG (collapsed SC) was the leading effect in HMPOAG eyes, and SC collapsed in the HMPOAG group as well as the POAG group. Thus, the morphology of SC was abnormal in both the POAG and HMPOAG groups. The collapse of SC could increase the aqueous humor outflow resistance and IOP, resulting in glaucomatous damage (17, 19, 21, 38). In addition to the collapse of SC, the obstruction of intra-scleral collector channels and deep scleral plexus caused by HM might further elevate the aqueous humor outflow resistance in the HMPOAG group, making its outflow resistance higher than that of POAG.

A recent study reported a mild correlation between temporal/nasal AST0 and ganglion cell layer (GCL) thickness in the HMG group and suggested that AST0 might be a novel indicator to discriminate glaucoma from healthy in HM subjects (34). In our study, we did not collect GCL thickness data. Thus, we only performed a correlation analysis between RNFL thickness and AST. The results showed that RNFL thickness was significantly and positively associated with all AST parameters (AST0, AST1, AST2, and AST3) in the HMPOAG group. Accordingly, based on the previous study (34) and our present results, AST was suggested to have associations with both RNFL and GCL thicknesses in the HMG group. Considering that RNFL and GCL thicknesses could be used for myopia with and without glaucoma discrimination (42), AST could be a potential clinical indicator to discriminate myopic eyes with and without glaucoma.

This study has certain limitations. First, we did not include normal controls in this study for comparison. Second, all of the subjects in the study were Chinese. Previous studies have reported ethnic differences in the AST. The AST of Caucasians was thinner than that of non-Caucasians (35). Thus, it is unclear whether similar results would be observed in other ethnic groups. Third, we only collected RNFL data, but no GCL data for further data analysis (34). Fourth, most of our study subjects received prostaglandin analog treatment. As a previous study indicated, prostaglandin analogs could have a potential effect on AST (43). However, further longitudinal studies are required to verify and quantify this effect (34).

## Conclusion

In conclusion, the HMPOAG group has the thinnest AST, shortest SS, thinnest TM, and smallest SC. The thinnest AST might contribute to the shortest SS, followed by the thinnest TM and smallest SC in the HMPOAG group. In addition, Kudsieh et al. (34) also suggested that distinct from parameters related to AL (e.g., anterior chamber depth and anterior chamber angle), AST might be an independent factor that is unaffected by AL. They implied that AST was more closely related to glaucoma than other parameters. Thus, AST might be a novel clinical indicator in the prediction and evaluation of POAG. In addition, due to the more abnormal morphology of TM and SC (the thinnest TM and smallest SC) caused by the thinnest AST and shortest SS length in the HMPOAG group, we speculated that the outflow resistance of HMPOAG eyes might be higher than that of both HM and POAG eyes. Previous studies have indicated that the progression of visual field loss was more significant in HMPOAG patients (8, 9), and the supposed reasons could be the thinner lamina cribrosa (44, 45), the thinner posterior sclera (46, 47), and the reduced ocular blood supply (32, 33, 48). In addition to these factors, the increased aqueous humor outflow resistance caused by the abnormal ocular anterior segment morphology (thinnest AST, shortest SS length, and the following thinnest TM, smallest SC) in the HMPOAG group might also be one potential reason for this phenomenon.

## Data availability statement

The raw data supporting the conclusions of this article will be made available by the authors, without undue reservation.

## Ethics statement

The studies involving humans were approved by the ethics committee of Tongji Hospital, Huazhong University of Science and Technology (TJ-IRB20201024). The studies were conducted in accordance with the local legislation and institutional requirements. The participants provided their written informed consent to participate in this study.

## Author contributions

ML: Conceptualization, Data curation, Formal analysis, Funding acquisition, Investigation, Methodology, Project administration, Resources, Software, Writing – original draft. LC: Data curation, Formal analysis, Investigation, Methodology, Resources, Software, Writing – original draft. ZL: Data curation, Formal analysis, Investigation, Methodology, Resources, Software, Writing – original draft. XY: Conceptualization, Formal analysis, Investigation, Project administration, Resources, Supervision, Validation, Visualization, Writing – review & editing.

## Footnotes

^1^http://www.r-project.org
